# Water system is a controlling variable modulating bacterial diversity of gastrointestinal tract and performance in rainbow trout

**DOI:** 10.1371/journal.pone.0195967

**Published:** 2018-04-17

**Authors:** Omolola C. Betiku, Carl J. Yeoman, T. Gibson Gaylord, Benjamin Americus, Sarah Olivo, Glenn C. Duff, Wendy M. Sealey

**Affiliations:** 1 United States Fish and Wildlife Service, Bozeman Fish Technology Center, Bozeman, Montana, United States of America; 2 Department of Animal and Range Science, Montana State University, Bozeman, Montana, United States of America; 3 Department of Animal and Range Sciences, New Mexico State University, Las Cruces, New Mexico, United States of America; Universitat Politècnica de València, SPAIN

## Abstract

A two-phase feeding study evaluating performance of rainbow trout and comparing luminal and mucosal gastrointestinal tract (GIT) bacterial community compositions when fed two alternative protein diets in two rearing systems was conducted. Alternative protein diets (animal protein and plant protein diets) balanced with crystalline amino acids: lysine, methionine and threonine or unbalanced, were fed to rainbow trout in two separate water systems (recirculating (RR) and flow-through (FF)) for a period of 16 weeks. The four diets, each contained 38% digestible protein and 20% fats, were fed to rainbow trout with an average weight of 12.02 ± 0.61 g, and sorted at 30 fish/tank and 12 tanks per dietary treatment. Phase 1 lasted for 8 weeks after which fish from each tank were randomly divided, with one-half moved to new tanks of the opposing system (i.e. from RR to FF and vice versa). The remaining halves were retained in their initial tank and system, and fed their original diets for another 8 weeks (phase 2). After the 16^th^ week, 3 fish/tank were sampled for each of proximate analysis, body indexes and 16S rRNA analysis of GIT microbiota. Fish weight (P = 0.0008, P = 0.0030, P<0.0010) and body fat (P = 0.0008, P = 0.0041, P = 0.0177) were significantly affected by diet, diet quality (balanced or unbalanced) and system, respectively. Feed intake (P = 0.0008) and body energy (P<0.0010) were altered by system. Body indexes were not affected by dietary treatment and water systems. Compositional dissimilarities existed between samples from the rearing water and GIT locations (ANOSIM: (R = 0.29, P = 0.0010), PERMANOVA: R = 0.39, P = 0.0010), but not in dietary samples (ANOSIM: R = 0.004, P = 0.3140, PERMANOVA: R = 0.008, P = 0.4540). Bacteria were predominantly from the phyla *Proteobacteria*, *Firmicutes* and *Bacteroidetes*. Their abundance differed with more dissimilarity in the luminal samples (ANOSIM: R = 0.40, P = 0.0010, PERMANOVA: R = 0.56, P = 0.0010) than those from the mucosal intestine (ANOSIM: R = 0.37, P = 0.0010, PERMANOVA: R = 0.41, P = 0.0010). Bacteria generally associated with carbohydrate and certain amino acids metabolism were observed in the mucosal intestine while rearing water appeared to serve as the main route of colonization of *Aeromonas* and *Acinetobacter* in the rainbow trout.

## Introduction

Host-microbial symbioses are vital to life and can be commensal, pathogenic, mutualistic or parasitic [1]. In vertebrate species, the complex microbial ecosystem in the gastrointestinal tract (GIT) plays a specific and important role in the physiology and metabolism of the host [2, 3]. Composition of the GIT bacteria are crucial to maintenance of host’s health and minor perturbations may lead to a shift in the microbial population, which may affect normal functioning of the host system. Most information on vertebrate gut microbiota has been primarily focused on mammalian species [4], with little attention on the lineage of fishes that represent over half of all vertebrate species [5]. Consequently, processes that underline the role of individual GIT microbes in nutrition and health of fish are still poorly understood.

Early cultivation-independent descriptions of the GIT microbiota in fishes have recently emerged [6, 7]. But what constitutes ‘core’ microbiota in aquatic animals has not yet been defined due to differences in bacterial communities reported in the literature. It also remains to be determined how environmental factors shape the composition of fish GIT microbiota. This information is important as maintaining a healthy microbiota is likely to improve performance of cultured fishes in aquaculture. Available evidence has shown that diet [8–11], rearing water [12], and combination of diet, water environment, feeding habitat [13], temperature and season [14] are important factors that modulate the normal GIT microbiota in fishes. While most fish microbiota studies have been directed towards characterization of the communities of microbes in the GIT lumen [10, 15–17], those that adhere to the mucosal surface, which are generally believed to be important in specialized physiological functions, remain uncharacterized.

Studies have proposed that core GIT microbiota in fishes are closely related to that of the fish feed or the rearing habitat [18], implying a largely allochthonous microbiota. This information for fish husbandry connotes that regulation of the external environment may be important for improving health and production of aquaculture fish species. This becomes essential when complete replacement of fishmeal is the goal for production of economically important fishes such as rainbow trout with a production sales of 104 million dollars in 2015 in North America [19]. Earlier studies of the 16S rRNA gene composition of rainbow trout GIT [15, 17] suggest the possibility of colonization by microbiota from the environment prior to first feeding and subsequently, less significant changes due to diets. However, it is difficult to ascertain whether feeding rainbow trout a fishmeal-free diet will have the same response on the microbiota composition of the GIT since the diets used in the studies by Desai et al. [15] and Ingerslev et al. [17] contained significant amounts of fishmeal as protein source, but Zarkasi et al. [20] demonstrated in an *in vitro* study that different dietary treatments changes the dynamics of the microbial community in hindgut of salmon.

Therefore, the present study investigated the performance of rainbow trout fed two fishmeal-free diets (animal- and plant-based diets) and assessed the impact of feeding these diets on the bacterial diversity and composition of the rainbow trout GIT. It also focused on characterization of the GIT bacteria associated with the luminal and the mucosal sections of rainbow trout GIT and establishment of the different bacteria that exist between trout GIT and the two commonly used water environments (recirculating and flow-through systems) in fish farming.

## Materials and methods

### Ethics statement

This present study was carried out in strict accordance with the guidance for the Care and Use of Laboratory Animals of the U.S. Fish and Wildlife Service (USFWS). The protocol was approved by the USFWS. Fish were euthanized by tricane methanesulfonate (MS-222) anesthesia with conscious efforts to limit the pain, trauma and distress of the fish.

### Experimental design for the study

A 2 x 2 x 2 factorial design experiment was conducted using two experimental diets: animal protein diet (APD) and plant protein diet (PPD) with two diet qualities: amino acid balanced and unbalanced, which were fed to trout in two different water systems: recirculating and flow-through. Four diets were formulated at 38% digestible protein and 20% lipids (Table 1), supplemented with or without free amino acids: lysine, methionine and threonine to meet the reported nutrient requirements for rainbow trout [21]. All fish were handled and treated in accordance with guidelines approved by the USFWS.

**Table 1:** Ingredients and chemical compositions of experimental diets.

Ingredient index	APD as-fed (g/kg)	Unbalanced APD as-fed (g/kg)	PPD as-fed (g/kg)	Unbalanced PPD as-fed (g/kg)
**Ethanol yeast**^1^	65.2	65.2	65.2	65.2
**Soybean meal**^2^	6.5	6.5	6.5	6.5
**Feather meal**^3^	73.7	73.7	0.0	0.0
**IDF chicken 42**^3^	72.9	72.9	0.0	0.0
**Blood meal US**^4^	44.9	44.9	0.0	0.0
**Soy protein concentrate**^2^	95.7	144.3	308.5	361.7
**Corn protein**^4^	95.7	95.7	95.7	95.7
**Wheat midds**^3^	56.2	56.2	56.2	56.2
**Poultry fat**^3^	0.00	0.00	13.6	13.6
**Wheat flour**^4^	193.2	195.4	159.3	155.3
**Menhaden fish oil**^5^	176.0	175.1	175.0	175.0
**Lecithin**	10.0	10.0	10.0	10.0
**Stay-C 35**	15.0	15.0	15.0	15.0
**Vitamin premix ARS 702**^6^	10.0	10.0	10.0	10.0
**TM ARS 640**^7^	10.0	10.0	10.0	10.0
**NaCl**	2.8	2.8	2.8	2.8
**Magnesium oxide**	0.6	0.6	0.6	0.6
**Potassium chloride**	5.6	5.6	5.6	5.6
**Monocalcium phosphate**	33.0	31.0	30.0	28.0
**Choline Cl 50%**	10.0	10.0	10.0	10.0
**DL-methionine**	7.6	0.0	7.5	0.0
**Lysine HCl**	29.4	0.0	28.6	0.0
**Threonine**	7.6	0.0	8.4	0.0
**Taurine**	5.0	5.0	5.0	5.0
**Yttrium oxide**	1.0	1.0	1.0	1.0
**Astaxanthin**	0.8	0.8	0.8	0.8
***Proximate composition***				
**Crude protein (g/kg)**	454.2	447.2	441.3	440.8
**Fat (g/kg)**	140.3	167.9	132.0	135.9
**Gross energy (MJ/kg)**	23.6	23.7	23.1	22.7

^1^ Archer Daniels Midland (Decatur, IL, USA).

^2^ Nelson & Sons Inc. (Murray, UT, USA).

^3^ MGP Ingredients, Inc. (Atchison, KS, USA.

^4^ Gavilon LLC, (Omaha, NE, USA).

^5^MGP Ingredients, Inc. (Atchison, KS, USA).

^6^Contributed per kg of diet: vitamin A (as retinol palmitate), 30,000 IU; vitamin D_3_, 2160 IU; vitamin E (as DL-%-tocopheryl-acetate), 1590 IU; niacin, 990 mg; calcium pantothenate, 480 mg; riboflavin, 240 mg; thiamin mononitrate, 150 mg; pyridoxine hydrochloride, 135 mg; menadione sodium bisulfate, 75 mg; folacin, 39 mg; biotin, 3 mg; vitamin B_12_, 90 μg.

^7^Contributed in mg/kg of diet: zinc, 37; manganese, 10; iodine, 5; copper, 3; selenium, 0.4.

### Fish culture

Rainbow trout eggs were obtained from Troutlodge Inc., Sumner, Washington, USA. The fish were cultured at the Bozeman Fish Technology Center, Bozeman, MT to fingerlings size before the commencement of the experiment. Prior to the start of the feeding trials, fish were acclimatized to tanks for one-week period. Thereafter, diets were randomly assigned to each of the tanks and fish were fed twice daily to apparent satiation for the 16-week period of the experiment. The 16-week period is divided into two phases: phase 1 (0–8 weeks) and phase 2 (8–16 weeks). For phase 1, fish with an average initial weight of 15.7 ± 0.41 g were randomly selected and stocked at the rate of 30 fish/tank into 12 recirculating and 12 flow-through tanks, with three replicate tanks per each of the four dietary treatments. Recirculating culturing water temperature was maintained at 15 °C, while flow-through culturing water temperature ranged between 14.2 and 15.5 °C. During phase 2, an additional 12 tanks were created in both the recirculating and flow-through culturing systems, making 24 culturing tanks in both water systems. Half of the fish in each of the tanks from phase 1 were randomly selected and moved to the other water system, (i.e. from recirculating to flow-through and vice-versa), while the remaining half were maintained in the same tank and system till the end of phase 2.

### Diet manufacturing

All ingredients for each of the diet were ground with an air swept pulverizer (model 18-H; Jacobson, Minneapolis, MN, USA). All the dietary treatments were pre-cooked using cooking extrusion (DNDL-44, Buhler AG, Uzwil, Switzerland) with an 18-s exposure to an average of 127 °C in the extruder barrel section. The die plate was water-cooled to an average temperature of 60 °C. Depending on the diet, pressure at the die head was varied from 15 to 30 bar. The 3-mm pellets produced were then dried in a pulse-bed drier (Buhler AG, Uzwil, Switzerland) for 25 min at 102 °C with a 10-min cooling period. The final moisture contents in all the diets were <10%. Thereafter, oil was added to the diets by top-coating.

### Digestibility determination

Standard methods (AOAC, 1995) were used for analysis of dry matter and ash of ingredients, diets and feces. Apparent digestibility coefficients (ADCs) were determined following the method of Cho et al. [22]. Yttrium oxide was used as an indigestible marker and 1% was added to each of the dietary treatments. Fecal samples were collected from the fish in each tank at the end of each phase and freeze-dried overnight at -50 °C. Yttrium and phosphorus were determined in diets and feces by inductively coupled plasma atomic absorption spectrophotometry (Bozeman Fish Technology Center Analytical Laboratory, Bozeman, MT).

### Fish sampling

Random sampling of ten fish from the initial fish population was carried out for determination of initial whole body proximate composition. Three fish were randomly selected from each tank and were euthanized by overdose of tricane methane sulfonate (MS-222; 325 mg/L water) following the approved protocol of the USFWS. Twelve hours post-feeding, three fish per tank (n = 3 per tank) were euthanasia and within 10 min, they were dissected ascetically to obtained both luminal and mucosal samples from the fish gut. Samples were frozen rapidly in liquid nitrogen and were stored at -20 °C until further processing. Water samples (in three replicates) were collected per tank using 50 ml sterile conical tubes at 8-week and 16-week periods. They were freeze and stored immediately at -20 °C until the time for DNA preparation. During each 8-week period, fish were weighed monthly and feed intake, feed conversion ratio (FCR), and weight gain were obtained per tank. At the end of each 8-week feeding phase, three fish from each tank were randomly sampled for determination of whole body composition; three additional fish were sampled from each of the tank for determination of viscerosomatic index (VSI), hepatosomatic index (HSI) and filet ratio (FR). In addition, three fish were randomly selected from each of the tank and dissected for collection of luminal and mucosal samples as earlier described.

### Sample amplification and sequencing

Water samples from each tank were filtered using 0.22 μl sterile Whatman filter, which was followed by DNA extraction using the MoBio PowerWater DNA isolation kit. While luminal and mucosal samples were pooled per tank (n = 3 replicates per diet), DNA was extracted using MoBio PowerFecal DNA Isolation kit. The manufacturer’s instructions were followed except for a 3-min bead-beating step instead of a 10-min vortex for the water samples. Variable regions 3 and 4 of the 16s rRNA genes were amplified using custom primers that included indexes to identify samples after sequencing. The PCR reaction ran for 30 cycles at 94 °C for 20 s, 52 °C for 30 s, and 72 °C for 45 s, using barcoded primers (SeqF(1–8) 5’AATGATACGGCGACCACCGAGATCTACAC(adaptor)-Index2(one of eight different 8nt codes used to distinguish among samples when pooled for sequencing)-TATGGTAATT(sequencing primer pad)-AT(linker)-CCTACGGGAGGCAGCAG (341fprimer)-3’ and SeqR(1–12) 5’-CAAGCAGAAGACGGCATACGAGAT(adaptor)-Index1(one of twelve different 8nt codes used to distinguish among samples when pooled 67 for sequencing)- AGTCAGTCAG(sequencing primer pad)-CC(linker)-GGACTACHVGGGTWTCTAAT (806r primer-3’). Amplicons were quantified using an Agilent 2200 tape station (Agilent, Santa Clara, CA, USA) and pooled at an equimolar concentration. Pooled amplicons were purified from residual PCR reagents and non-specific amplification products in an agarose gel using a QIAquick gel extraction kit (Qiagen, Valencia, CA, USA) following manufacturer’s instructions. Purified and pooled amplicons were subsequently quantified using a KAPA Syber quantification kit (KAPABiosystems, Wilmington, MA, USA) as per manufacturer’s instructions. Purified, quantified and pooled amplicons were mixed with 10% of an equimolar concentration of PhiX. Sequencing was performed with an Illumina MiSeq using paired-ended 2 by 250 nucleotide (nt) dual-index sequencing. Custom primers (R1 5’-CCTACGGGAGGCAGCAG-3’, R2 5’-AGTCAGTCAGCCGGACTAC-3’, Index 5’-GTAGTCCGGCTGACTGACT-3’) were used for sequencing and indexing.

### Sequence processing and statistical analyses

Raw sequence data were filtered to remove low quality sequences (<Q30) and the remaining sequences were binned using USEARCH into operational taxonomic units (OTUs) at a threshold of over 97% sequence identity. To determine the identity or closest relative of the bacteria isolated from rainbow trout intestinal tract, we analyzed the 16S rRNA gene from this study against the public Ribosomal Database Project ll. OTUs with a relative abundance of < 0.05% were removed. Alpha diversity of level 0.05 was performed on the OTUs and Chao 1 and Simpson indices were obtained using the RAM package [23] in R. Venn diagram was constructed by jvenn tool [24]. Non-parametric analyses, including analysis of similarities (ANOSIM), permutation multivariate analysis of variance (PERMANOVA) and canonical analysis of principal (CAP) coordinates were performed using the PRIMER-E software (Plymouth, UK), and were used to explain a significant proportion of the total variation that exist in in our data. PERMANOVA was performed with 999 permutations, while CAP, a non-parametric permutation test, identified whether bacterial abundance differ among the treatment groups. P-values for the analyzed variables were corrected for false discovery rate [FDR (*Q*>0.05)] using R statistical software. The growth performance data were analyzed for factorial analysis of variance (ANOVA) using Proc GLM of SAS version 9.4 (SAS Institute Inc., Cary, NC, USA). Tukey’s means separations were used to determine differences within main effects. Significant effects were considered at P<0.05.

## Results

### Diets proximate compositions and digestibility

Proximate compositions of the diets manufactured in this study reflected the dry matter, crude protein and crude fat for balanced APD, unbalanced APD, balanced PPD and unbalanced PPD diets (Table 1). The apparent digestibility coefficients are presented in Table 2.

**Table 2:** Chemical analysis (% dry weight) and apparent digestibility coefficients (ADCs) of nutrients in unbalanced or balanced APD and PPD.

Item (%)	APD	Unbalanced APD	PPD	Unbalanced PPD	P>F^2^
**Dry matter**	66.7	66.2	64.8	62.3	0.0136
**Crude protein**	86.2	86.4	88.4	90.0	<0.0010
**Crude fat**	82.1	87.0	83.1	85.7	<0.0010

### Growth performance, body condition indexes and compositions of rainbow trout

Following the 16-week feeding trial, fish survival was within 97–100% for all dietary treatments. Average fish weight was significantly affected by diet (P = 0.0008) and diet quality (P<0.0030; Table 3).

**Table 3:** Growth performance and whole-body (WB) composition of rainbow trout fed animal and plant protein diets^1^.

Performance	Diet (D)		Diet quality (DQ)		Diet x diet quality (DDQ)				System (S)				P>F^2^					SEM^2^
	APD	PPD	Balanced	Unbalanced	Balanced		Unbalanced		FF	FR	RR	RF	Diet	DQ	DDQ	S	D x S	
					APD	PPD	APD	PPD										
**Final weight (g)**	120.12	111.27	119.52	111.86	123.20	115.85	117.04	106.68	102.82	119.75	117.67	122.5	0.0008	0.0030	0.5323	<0.0001	0.2284	4.62
**Feed intake**^3^ **(% bw/d)**	2.02	1.96	1.96	2.02	1.98	1.93	2.05	1.99	2.04	2.10	1.93	1.88	0.1508	0.0991	0.9106	0.0008	0.7140	0.07
**FCR**^4^ **(g feed/g gain)**	1.03	1.03	1.01	1.05	1.00	1.01	1.04	1.06	1.08	0.98	1.00	1.04	0.4286	0.0061	0.7910	0.0003	0.8523	0.03
**Condition indices**																		
**VSI**^5^ **(%)**	11.06	11.23	11.11	11.19	11.10	11.12	11.03	11.35	12.27	10.87	9.94	11.52	0.5273	0.7690	0.5682	<0.0001	0.6734	0.54
**FR**^6^ **(%)**	26.70	26.33	26.95	26.08	27.48	26.43	25.92	26.23	25.81	27.10	26.42	26.75	0.3357	0.0271	0.0829	0.1199	0.0421	0.76
**HIS**^7^ **(%)**	1.38	1.48	1.30	1.57	1.29	1.30	1.48	1.67	1.27	1.32	1.28	1.86	0.6233	0.1821	0.6525	0.1247	0.9619	0.40
**Whole-body Composition**																		
**Fat (g/kg)**	118.1	110.1	110.8	117.5	114.0	122.2	107.5	112.7	117.9	108.4	113.2	116.8	0.0008	0.0041	0.4828	0.0177	0.3756	0.43
**Energy (MJ/kg)**	8.8	8.7	8.7	8.8	8.7	8.7	9.0	8.7	9.4	8.1	9.2	8.4	0.2538	0.1246	0.2942	<0.0001	0.0876	50.45
**Protein (g/kg)**	160.1	164.3	163.4	161.0	162.7	164.1	157.5	164.6	165.9	158.7	162.2	162.1	0.0629	0.2848	0.1974	0.1600	0.4028	0.44

FF—Flow-through system, FR—From Flow-through system to recirculating system, RR—Recirculating system, RF—From recirculating system to flow-through system.

^1^Means of three replicates per diet

^2^Pooled standard error of the mean

^3^Feed intake (% bw/d) = g dry feed consumed/average fish biomass (g) /culture days *100.

^4^FCR = g dry feed consumed / g weight gained.

^5^VSI (%) = viscera mass x 100 / fish mass

^6^FR (%) = fillet with rib mass * 100 / fish mass.

^7^HIS (%) = liver mass x 100 / fish mass.

Fish fed the APD diet grew bigger than those on PPD diet and fish fed balanced diets were bigger than those fed unbalanced diets. Feed intake was the same irrespective of dietary treatments (P = 0.1986) and balancing the diets by inclusion of crystalline amino acids had no significant effect on intake (P = 0.2801). In addition, balancing diets did not affect (P = 0.0657) feed utilization in trout. Dietary treatments (P = 0.9645) and diet quality (P = 0.7690) had no significant effect on viscerosomatic index. Likewise, hepatosomatic index (P = 0.6196, P = 0.2043), condition index (P = 0.9654, P = 0.8908) and fillet ratio (P = 0.9645, P = 0.8910) were not significantly affected by dietary treatments and diet quality, respectively. Analyzed whole body energy (P = 0.2538, P = 0.1247) and protein (P = 0.0629, P = 0.2849) showed no negative effect due to diet and diet quality, respectively. However, significant effects were observed in body fat (P = 0.0008, P = 0.0041) with diet and diet quality, respectively.

Trout raised in the recirculating system grew bigger (P<0.0001) than those raised in flow-through system and switching the fish from either system to the other had no significant effect on the growth of trout. Feed intake (P = 0.0008) was higher in fish raised in flow-through than those in the recirculating system, which affected feed utilization in same system (P = 0.0003). Raising trout in either recirculating or flow-through systems had no significant effect on hepatosomatic index (P = 0.1247), condition index (P = 0.3033) and fillet ratio (P = 0.1199). Fish raised in the flow-through system stored more fat in the gut compared to those raised in the recirculating system. Body energy (P<0.0001) and fat (P = 0.0177) were significantly higher in fish raised in the flow-through water. All water physicochemical properties measured in both water systems were within the normal range. Nitrate ranged from 0.04 ± 0.01–0.17 ± 0.01, with highest concentration observed in the recirculating water system. While temperature (15.27 ± 0.15–15.53 ± 0.06), ammonium (0.09 ± 0.02–0.12 ± 0.03) and dissolved oxygen (DO) (6.32 ± 0.30–7.02 ± 0.19) were not different between the recirculating and flow-through water systems.

### Characteristics of the bacterial community compositions

In total, 68,493,243 high quality sequences were obtained following quality trimming with 3,668 OTUs identified. A total of 253,425 sequences containing 15 OTUs, were shared across all samples with 95% sequence identity. Good’s coverage estimates indicated that >99% of all OTU were surveilled in this study. Overall, Chao1 diversity indexes and measures of effective number of species (ENS) indicated greater richness and diversity in water samples than those from the fish GIT (Tables 4 and 5) and a Kruskal-Wallis test suggested significant differences between the GIT samples and the water samples (P = 0.0016). Overall, bacterial richness and diversity varied between APD and PPD diets. Measures of microbial richness and diversity in the luminal and mucosal regions of the fish GIT approached significance but did not vary between APD and PPD diets (Wilcoxon rank test, P = 0.0724). Greater species richness was observed in luminal GIT samples than those from the mucosa. This effect was consistent across the two diets, but significance difference was observed only in mucosal samples from APD-fed fish and luminal samples from PPD-fed fish (Wilcoxon rank test, P = 0.0048).

**Table 4:** Bacterial diversity indexes of samples from trout fed animal and plant protein diets.

Gut Samples	Balanced	Unbalanced	Luminal	Mucosal	RR	RF	FF	FR
***APD-diet***								
**#sequence**	18339	18339	18339	18339	18339	18339	18339	18339
**Chao**	84.9	73.5	82.8	75.3	58.9	98.7	64.7	87.2
**Shannon**	3.18	2.84	3.03	2.97	2.94	3.25	2.47	3.31
**ENS**	24	17	21	20	19	26	12	27
**Evenness**	0.81	0.73	0.76	0.78	0.86	0.75	0.67	0.31
**Species number**	84.9	73.5	108.5	52.1	58.9	86.1	66.6	64.3
**Simpson**	0.9	0.81	0.84	0.86	0.87	0.86	0.75	0.92
***PPD-diet***								
**#sequence**	18339	18339	18339	18339	18339	18339	18339	18339
**Chao**	104.6	103.9	109.5	99.5	124	157.8	61.3	87.4
**Shannon**	3.45	3.26	3.42	3.29	3.52	3.82	2.95	3.23
**ENS**	32	26	31	27	34	46	19	25
**Evenness**	0.8	0.79	0.76	0.83	0.77	0.25	0.77	0.81
**Species number**	104.6	103.9	109.5	99.5	124	157.8	61.3	87.4
**Simpson**	0.3	0.26	0.89	0.9	0.91	0.92	0.85	0.91

FF—Flow-through system, FR—From Flow-through system to recirculating system, RR—Recirculating system, RF—From recirculating system to flow-through system.

**Table 5:** Comparison of bacterial diversity indexes of water samples from trout fed animal and plant protein diets.

Water Samples	Balanced	unbalanced	RR	RF	FF	FR
***APD-Diet***						
**#sequence**	18327	18327	18327	18327	18327	18327
**Chao**	118.2	105.9	152.8	77.5	70.3	133.7
**Shannon**	3.42	2.96	3.65	2.82	2.79	3.3
**ENS**	31	19	39	17	16	27
**Evenness**	0.73	0.65	0.75	0.66	0.66	0.69
**Species number**	118.2	105.9	152.8	77.5	70.3	133.7
**Simpson**	0.92	0.85	0.93	0.87	0.87	0.87
***PPD-Diet***						
**#sequence**	18327	18327	18339	18339	18339	18339
**Chao**	71.7	132.4	133	74.7	99.2	70.3
**Shannon**	3	3.6	3.5	3.11	3.2	3
**ENS**	20	36	33	22	25	20
**Evenness**	0.73	0.77	0.71	0.73	0.73	0.72
**Species number**	71.7	132.4	133	74.7	99.2	70.3
**Simpson**	0.91	0.93	0.93	0.91	0.91	0.91

FF—Flow-through system, FR—From Flow-through system to recirculating system, RR—Recirculating system, RF—From recirculating system to flow-through system.

### Bacterial community composition and importance of diet type and rearing water

**Fig 1:**
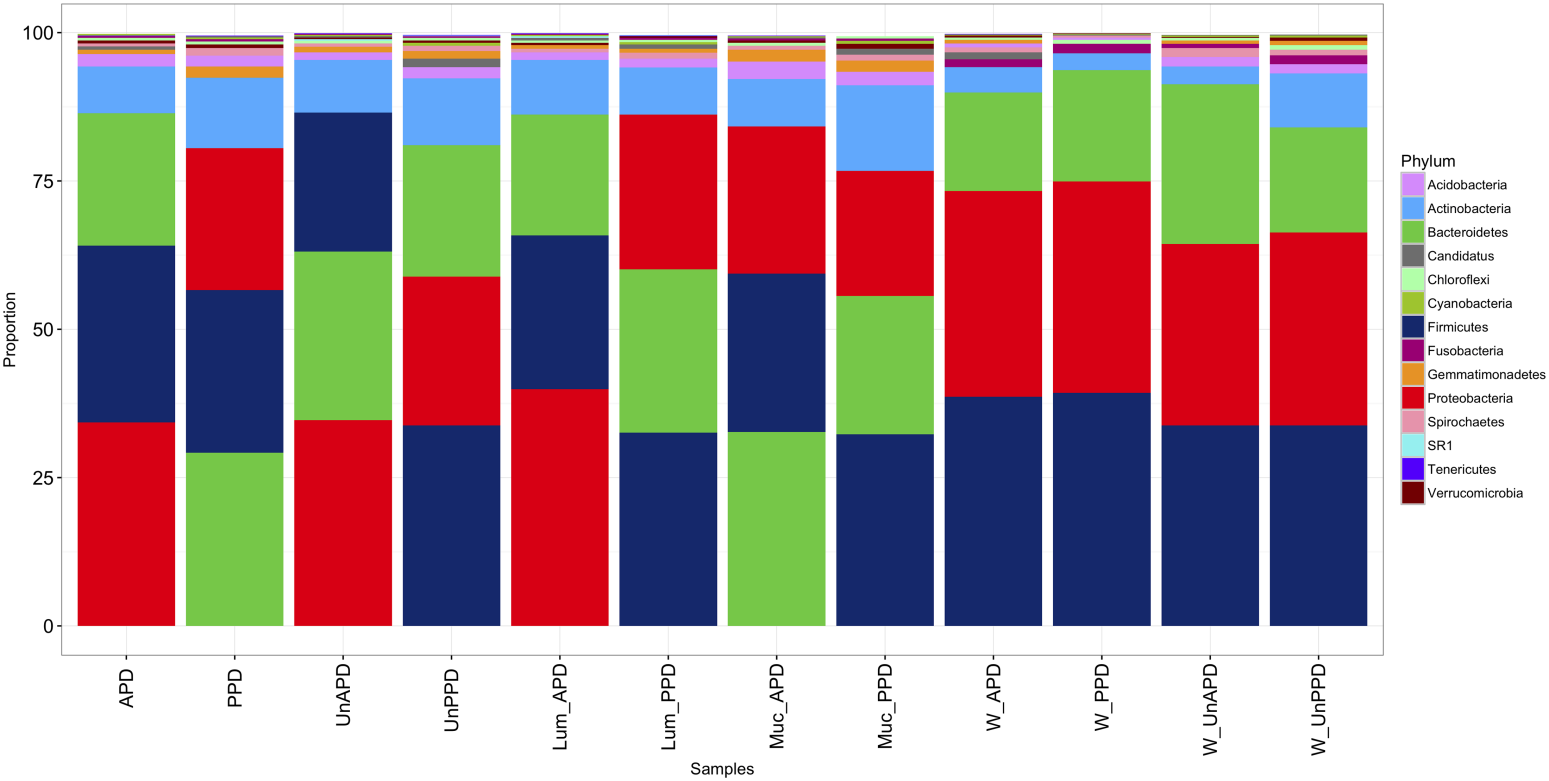
Bacterial composition at phylum level of rainbow trout fed animal and plant protein diets. Digesta and water samples are grouped by dietary treatments. Dietary treatments: Balanced Animal protein diet (APD), Unbalanced animal protein diet (UnAPD), Plant protein diet (PPD),Unbalanced plant protein diet) (UnPPD), Luminal samples by Balanced APD diet (Lum_APD), Luminal samples by Balanced PPD diet (Lum_PPD), Mucosal samples by Balanced APD diet (Muc_APD), Mucosal samples by Balanced PPD diet Muc_PPD); and Water samples: Samples from APD diet (W_APD), Samples from PPD diet (W_PPD), Samples from Unbalanced APD diet (W_UnAPD), Samples from Unbalanced PPD diet (W_UnPPD).

Bacteria were predominantly of the *Proteobacteria*, *Firmicutes*, *Bacteroidetes*, *Actinobacteria* and *Spirochaetes* phyla (Fig 1) and their relative abundances varied among water, luminal and mucosal GIT samples. Generally, GIT samples from this study contained 31% *Firmicutes*, 31% *Proteobacteria*, 27% *Bacteroidetes*, 10% *Actinobacteria* and 2% *Acidobacteria*, while water samples slightly differed with 38% *Firmicutes*, 34% *Proteobacteria*, 22% Bacteroidetes, 5% *Actinobacteria*, and 1.1% *Acidobacteria*. CAP coordinates showed overall significance of all the OTU abundance data (*R*^2^ = 0.94) in this study.

**Fig 2:**
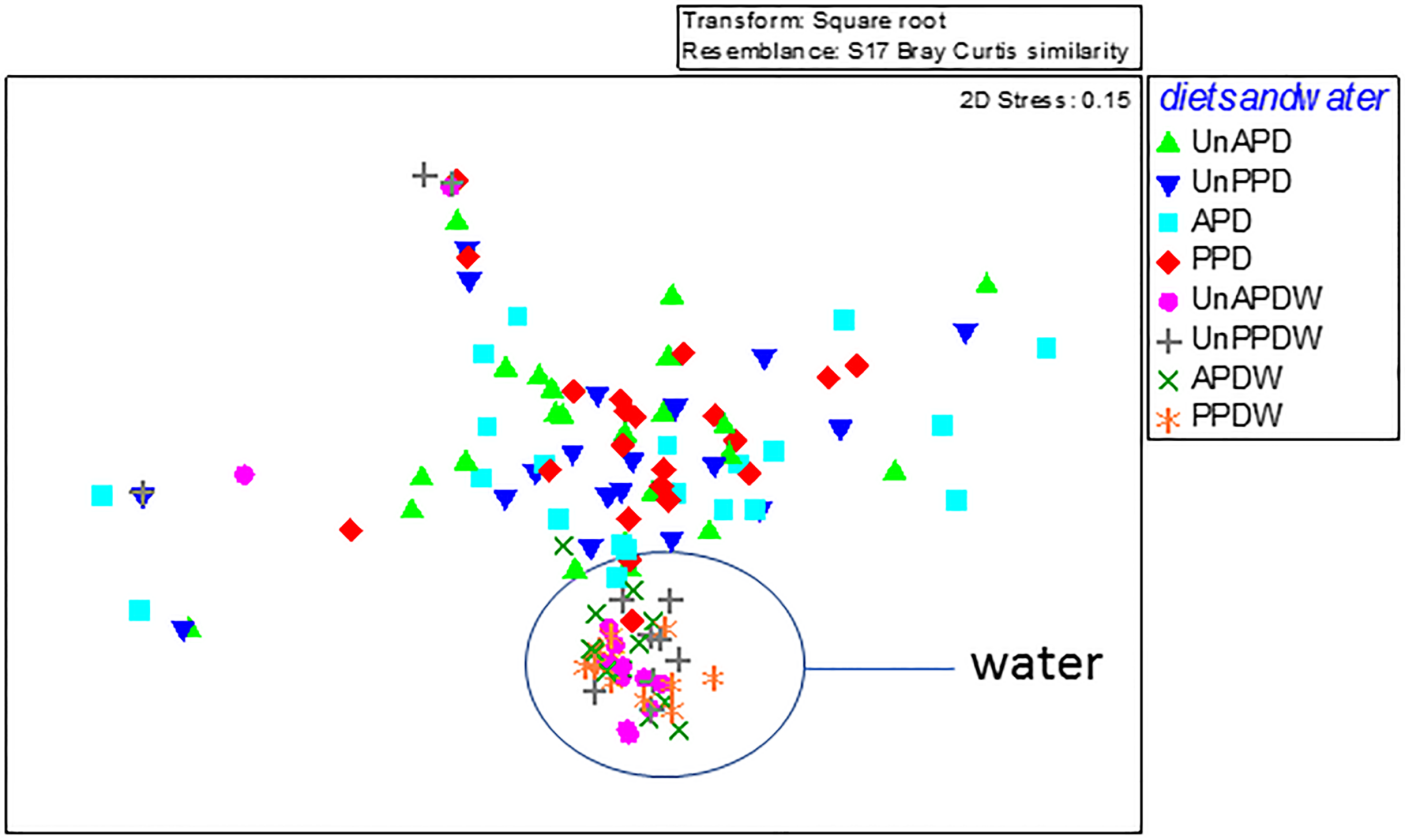
Non-metric multidimensional scaling plot based on Bray-curtis similarity of bacterial communities from fish gut and water samples. Digesta samples: Balanced animal protein diet (APD), Balanced plant protein diet (PPD), Unbalanced animal protein diet (UnAPD), Unbalanced plant protein diet (UnPPD); water samples: Balanced animal protein diet (APDW), Balanced plant protein diet (PPDW), Unbalanced animal protein diet (UnAPDW), and Unbalanced plant protein diet (UnPPDW).

Estimations of the relative magnitude of our treatments on microbial β-diversity indicated that water alone produced a higher positive correlation (*R*^2^ = 0.83) than diets (*R*^2^ = 0.55), which indicated that trout GIT microbiota shared greater similarity with the rearing water. Neither water nor GIT samples varied by dietary treatment (ANOSIM: R = 0.004, P = 0.3140, PERMANOVA: R = 0.0080, P = 0.4540). However, β-diversity differed between water samples and those obtained from trout mucosal and luminal GIT (ANOSIM: R = 0.29, P = 0.0010), PERMANOVA: R = 0.39, P = 0.0010) (Fig 2). Luminal samples had higher compositional dissimilarities (ANOSIM: R = 0.40, P = 0.0010, PERMANOVA: R = 0.56, P = 0.0010) compared to those from the mucosal region of the GIT (ANOSIM: R = 0.37, P = 0.0010, PERMANOVA: R = 0.41, P = 0.0010). Pairwise comparisons of all analyzed variables revealed only significant difference between mucosal and luminal samples from unbalanced APD diet (P = 0.0170) as well as mucosal samples from unbalanced APD and luminal samples from PPD diets (Table 6). For water samples, significant differences were observed between fish raised in the flow-through and recirculating systems, FF-RR (P = 0.0010); fish raised in the flow-through system and those moved from flow-through to recirculating system, FF-RF (P = 0.0040); fish raised in the recirculating system and those moved from recirculating to flow-through system, RR-FR (P = 0.0360) and fish moved from flow-through to recirculating system compared to those moved from recirculating to flow-through water systems, FR-RF (P = 0.0050).

**Table 6:** Results of PEMANOVA analysis for the different diets, gut locations, and water samples.

**PERMANOVA**	**P-value**	**FDR**			
**Diet**	0.1410	0.187			
**Diet quality**	0.3080	0.32			
**Water**	0.0030	0.011			
**Location**	0.0010	0.005			
**PERMANOVA pair wise test**	**P-value**	**FDR**	**PERMANOVA pair wise test**	**P-value**	**FDR**
**Gut locations**			**Water samples**		
**UnAPD**_**M**_**—PPD**_**L**_	0.0010	0.006	**FR, FF**	0.0430	0.065
**UnAPD**_**M**_**—UnAPD**_**L**_	0.0060	0.018	**FR, RR**	0.2960	0.32
**APD**_**M**_**—PPD**_**L**_	0.0610	0.0732	**FR, RF**	0.0040	0.013
**PPD**_**L**_**—PPD**_**M**_	0.0880	0.088	**FF, RR**	0.0010	0.005
**APD**_**M**_**—UnAPD**_**L**_	0.0220	0.044	**FF, RF**	0.0160	0.034
**PPD**_**M**_**—UnAPD**_**L**_	0.0370	0.0555	**RR, RF**	0.1550	0.191

**Fig 3:**
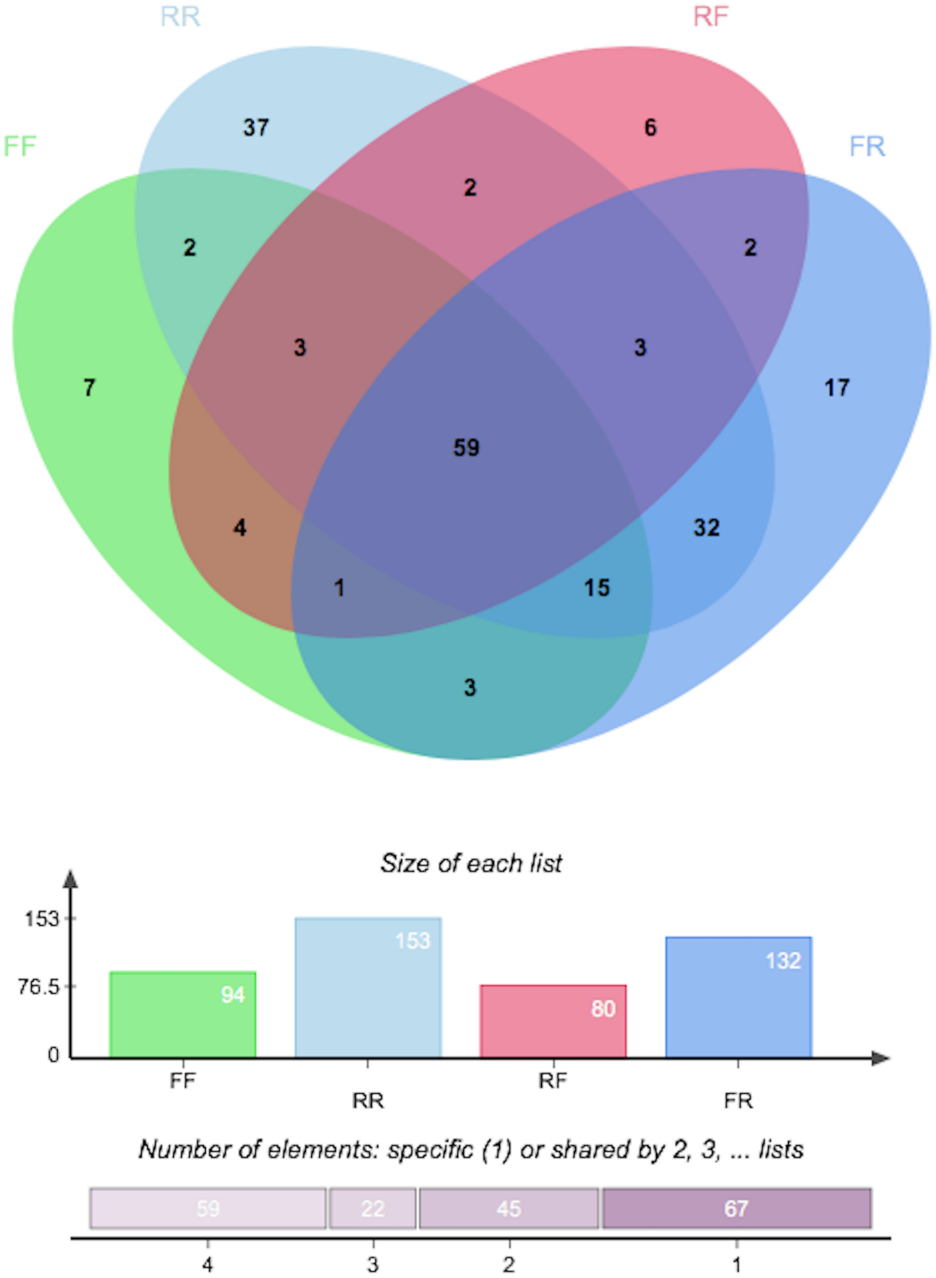
Venn diagram showing bacterial richness of trout GIT when reared in recirculating and flow-through water systems. RR: recirculating, FF: flow-through, FR: flow-through-recirculating, and RF: recirculating-flow-through.

Bacterial diversity of the GIT was greater in fish switched from the flow-through to recirculating system than fish switched from recirculating to flow-through system (Fig 3).

**Fig 4:**
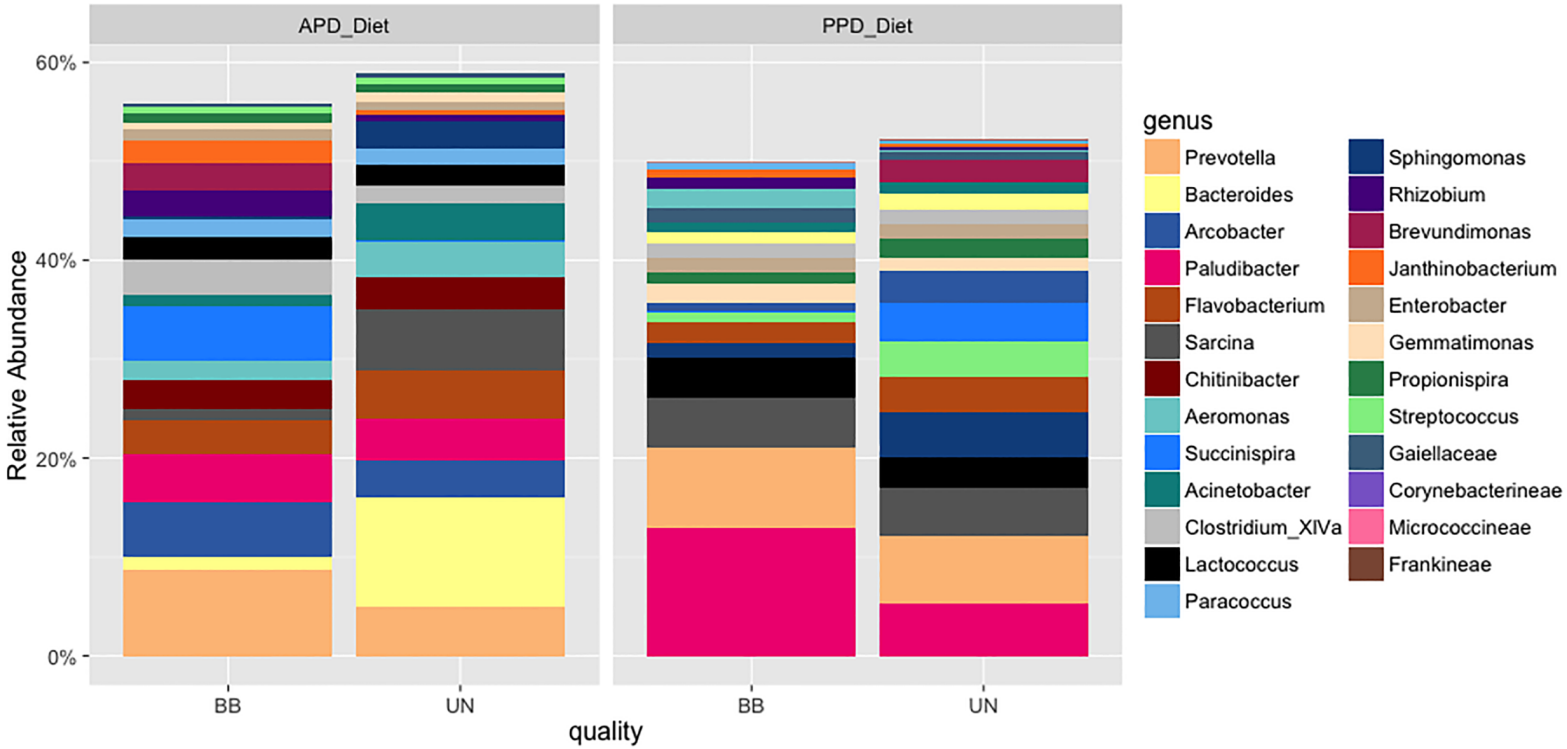
Bar chart of the relative abundance of bacterial community compositions at genus level at dietary treatments level with (BB) or without (UN) substitution with crystalline amino acids.

**Fig 5:**
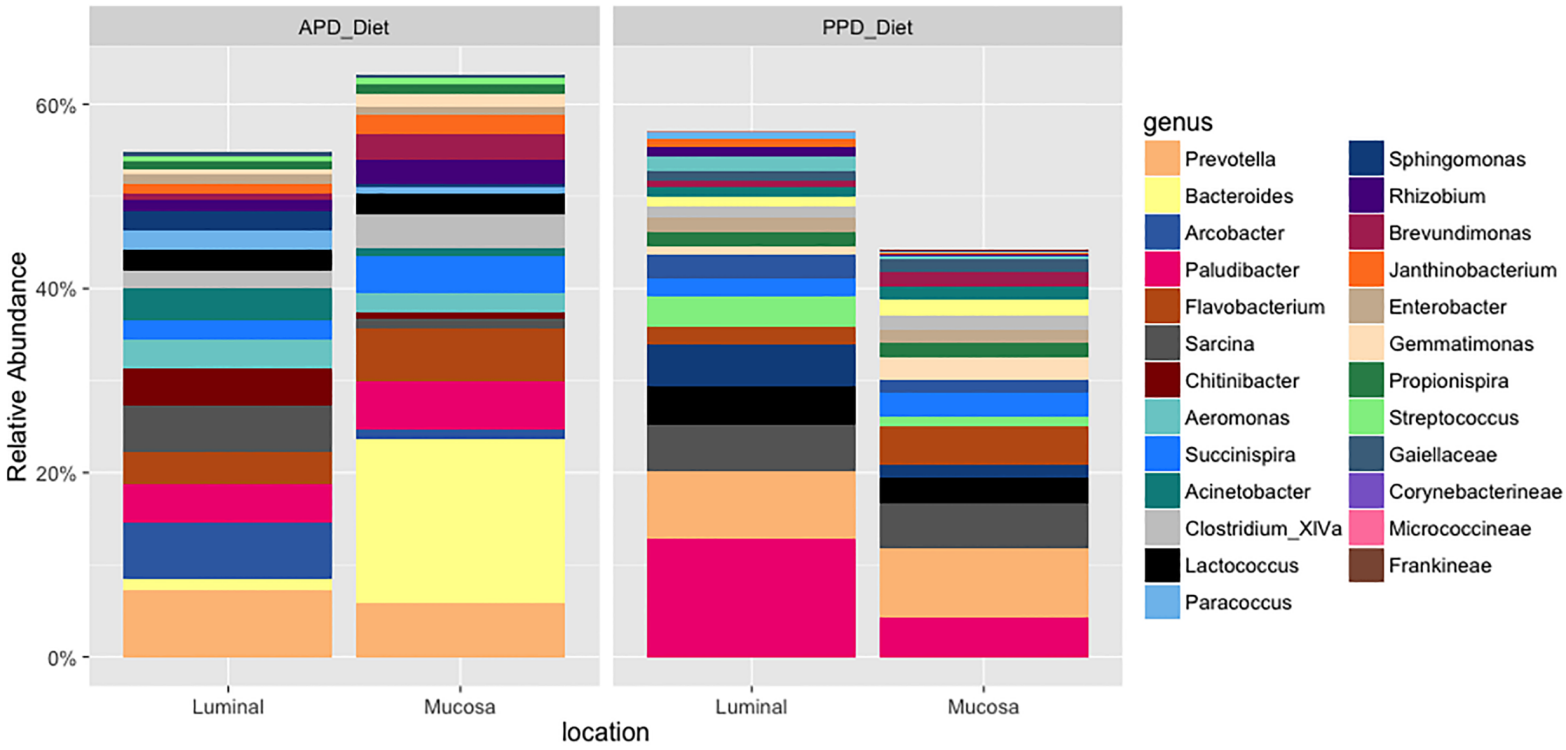
Bar chart of the relative abundance of bacterial community compositions at genus level at GIT locations.

**Fig 6:**
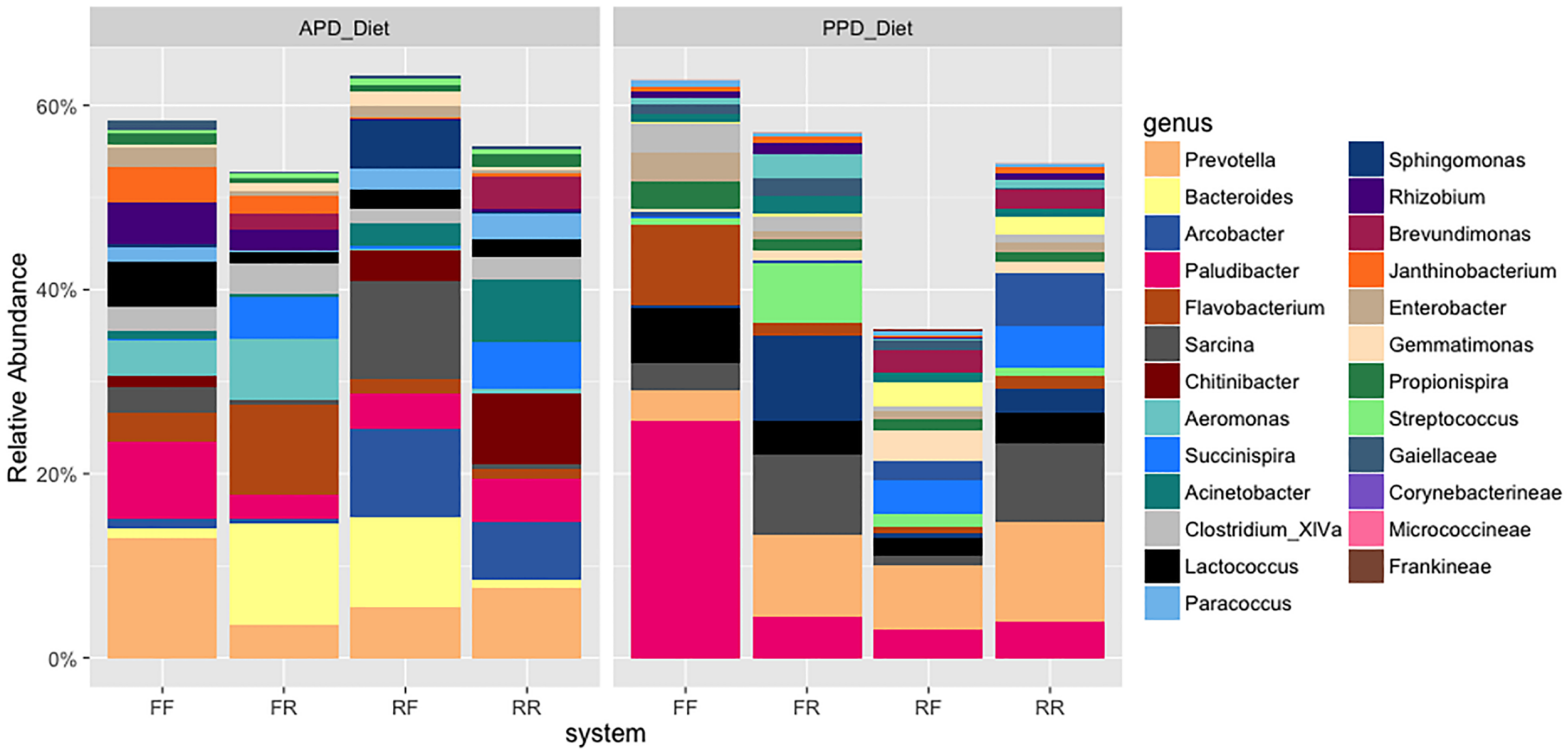
Bar chart of the relative abundance of bacterial community compositions at genus level at rearing water levels. Flow-through (FF), moved from flow-through to recirculating (FR), recirculating (RR) and moved from recirculating to flow-through (RF).

The major bacterial genera composition in the diets (Fig 4), GIT locations (Fig 5) and rearing water (Fig 6) were *Prevotella*, *Bacteroides*, *Arcobacter*, *Paludibacter*, *Aeromonas* and *Flavobacterium*, with enrichment of *Bacteroides* in the APD diet while *Paludibacter* was enriched in the PPD diet. Significant differences were observed in the relative abundances of 70 microbes between the mucosal and the luminal region of the GIT (S1 Fig), and each of the mucosal and luminal GIT samples and water samples (S2 and S3 Figs) (P < 0.05). The mucosal GIT had greater relative abundances of *Tolumonas*, *Chitinophaga*, *Anaerosalibacter*, *Coriobactineae*, *Peptoniphillus*, *Pseudobutyrivibrio propionivibrio*, *Enhydrobacter*, *Alcaligenes* and *Ethanoligenens* than observed in the luminal GIT samples (S1 Fig).

**Fig 7:**
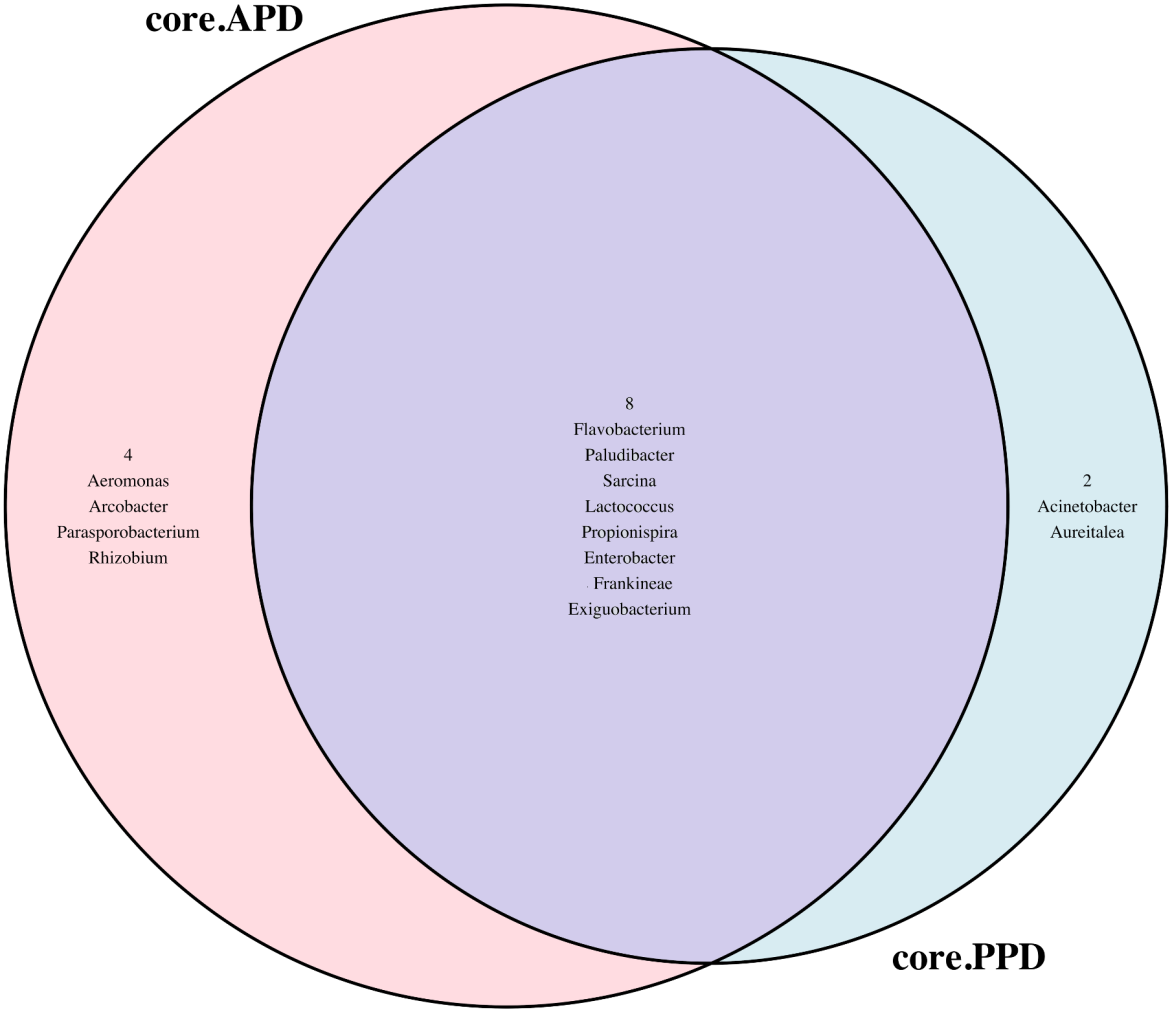
Venn diagram showing core bacteria associated with animal protein diet (APD) and plant protein diet (PPD).

Relative to water samples, *Arcobacter*, *Brevundimonas*, *Corynebacterineae*, *Sarcinia*, *Propionibacterineae*, *Exiguobacterium* and *Clostridium* were enriched in the trout mucosal region (S2 Fig), while *Enterobacter*, *Lactococcus*, *Paracoccus*, *Chitibacter*, *Succinispira and Gemmatimonas* were enriched in the luminal sample. (S3 Fig). *Parasporobacterium*, *Janthinobacterium*, *Flavobacterium*, *Carnobacterium*, *Acetanaerobacterium*, *Deefgea* and *Clostridium XIVa* exhibited greater relative abundances in water samples when compared to each GIT regions (S2 and S3 Figs). However, we did not observe significant separation of the bacterial composition between APD-fed fish and those fed PPD diet (Fig 7). Greater relative abundances of *Aeromonas* and *Arcobacter* were observed in APD-fed fish, while a greater relative abundance *Acinetobacter* was associated with PPD diet (Fig 7), but *Flavobacterium*, *Paludibacter*, *Lactococcus*, *Propionispira*, *Enterobacter* and *Exiguobacterium* were common in the GIT of both fed APD and PPD diets (Fig 7).

## Discussion

In the current study, we evaluated the performance of rainbow trout fed animal-and plant-protein diets and the effect of the diets were assessed on the bacterial composition within the luminal and mucosal sections of trout GIT. This study is the first to implement next-generation sequencing technology to investigate changes in the bacterial composition of rainbow trout luminal and mucosal intestinal sections when the fish is switched from recirculating water system to flow-through water system and vice versa.

Animal and plant protein diets used in this study had no detrimental effect on rainbow trout survival. This observation shows that trout can survive on animal and plant protein-based diets that are devoid of fishmeal when supplemented with the essential amino acids: lysine, methionine and threonine. Our findings corroborate an earlier report that rainbow trout utilizes free amino acids efficiently [25]. Differences were observed in the growth performance of fish fed the animal and plant protein diets, despite supplementation with the three most-limiting amino acids. Although diets were formulated on a digestible amino acid basis, we expected no growth differences between fish fed the two diets, given similar feed intakes for both animal and plant protein diets. The reduced growth observed in trout fed the plant protein diet may indicate less-bioavailability of certain amino acids that were not supplemented in the plant diet or other reductions in bioavailability of certain nutrients due to antagonistic interactions with antinutritional factors of the plant diet. For instance, zinc bioavailability has previously been shown to be affected with soybean meal diets leading to reductions in growth of rainbow trout [26].

In addition, slight increase in body fat observed in fish fed the plant protein diet may be as a consequence of non-starch polysaccharides in the plant ingredients affecting fat digestibility in the fish [27, 28]. These reductions in fat digestibility may have reduced the growth response from the fish. Low quality protein, especially from plant ingredients as observed from trout fed the unbalanced plant protein diet, is in accordance with what has been reported in the literature [25, 29]. Unbalanced amino acid profile of this non-supplemented plant protein diet may have resulted in the fish eating more, but unable to use it for protein accretion, instead catabolizing the amino acids for energy. Overall, there was no significant difference in trout gut bacterial composition due to feeding non-aquatic animal and plant protein-based diets. Although, earlier studies on rainbow trout reported that changes in microbial composition is due to diet effect [15, 17]. It is difficult to compare these earlier studies with our study since effect of rearing water on gut microbial population was not accounted for in those studies. However, report on Atlantic cod observed that water is a more major factor than diet in shaping gut microbiota in this fish [30].

In this current study, trout cultured in the recirculating system performed better than those raised in the flow-through system. This observation is supported by earlier findings on rainbow trout under these two water systems [31]. We hypothesize that the observed differences in microbial diversity possibly impacted trout performance. The observation of more bacterial species richness in the recirculating system than flow-through system may have contributed significantly to the better performance of the fish that were raised in the recirculating system. It has been suggested that the recirculating water system has properties that enhance microbial stabilization, owing to large surface area of the biofilters and more time for water retention compare to the flow-through system [32, 33]. Also, ability of the recirculating system to selectively secure a microbial maturation environment due to more microbial diversity and biomass in the recirculating system than flow through system was observed in cod larvae [34]. Our finding on improved performance of trout raised in recirculating system corroborated by earlier report on Atlantic cod larvae, which was attributed to more stable and diverse microbial community in recirculating system than the flow through system [35]. Although the contribution of bacteria in rearing water to host physiology is not known in fishes. Report on Indian white shrimp revealed increased activities of amylase, proteases and lipases associated with higher counts of *Bacillus* bacteria in the GIT of the treatment group when *Bacillus spp*. was added as probiotics into the water [36]. Similarly, improved growth performance was observed in *Macrobrachium rosenbergii* when different strains of lactobacillus were fed as probiotics compared to the control [37]. Thus, we postulated that more diverse microbial composition in recirculating system than flow-through system had significant impact on trout adaptation and performance in this study.

High abundances of *Proteobacteria*, *Firmicutes*, *Bacteroidetes* and *Actinobacteria* were observed in this study, consistent with what have previously been reported for rainbow trout [15, 17, 38–40] and Atlantic salmon [41, 42]. In particular, high abundances of the phylum *Proteobacteria* may reflect the ability of this group of bacteria to exhibit both host and environmental lifestyles [43]. Enrichment of *Bacteroides* from phylum *Bacteroidetes*, which is associated with high protein and fat diets in human studies [44, 45], observed in both the luminal, mucosal and the rearing water samples in this study especially APD diet, supports the notion that digestive physiology of trout has evolved utilizing protein and lipid for metabolic energy [46] and also the possibility of close proximity between microbial ecology of trout and higher vertebrates [46]. Our observation agrees with earlier findings that the mucosal region of the GIT is more diverse than the luminal section of trout gut [47]. Greater microbial diversity associated with the PPD diet may reflect a broadening of niches by the high fiber content of the plant protein-based diet. A previous study of Cichlids reported a reduction in microbial diversity among captive strains of Cichlids that were fed high protein and fat diets rather than the fiber-rich diets obtained in with low fiber diet [48].

The phylum *Acidobacteria*, generally associated with soil environments [49], observed both in the GIT and water samples, probably reflect a transient influence of the environment on the GIT. The genus *Flavobacterium* includes *F*. *psychrophilum*, a species that is the etiological agent of rainbow trout fry syndrome [50, 51]. *Flavobacterium* spp. were observed in luminal and mucosal samples of fish fed plant and animal protein diets and with higher relative abundance in the water sample than samples from the luminal and mucosal GIT. This finding is consistent with an earlier suggestion that the *Flavobacterium* lineage includes non-pathogenic species or isolates [52] and rearing water have been suggested as the medium by which viable cells are transmitted into the fish [53]. Higher enrichment of cellulose-degrading bacteria: *Brevundimonas*, *Anoxybacillus and Robinsoniella* [54, 55] as well as genus *Enhydrobacter* (a member of *Vibrionaceae* family), which are known to utilize certain amino acids *viz*. L- alanine, L-serine, and L-arginine for growth and Chitinolytric bacteria, showed relative importance of exogenous and endogenous digestive enzymes associated with the digestive systems of fishes, including rainbow trout [56–58]. *Chitinophaga* has always been commonly isolated from the intestinal tract of hatchery fed trout where they play a defense rather than digestive role [59]. We suggest further investigation of this role in rainbow trout health.

## Conclusions

In summary, our study investigated the importance of diet types and rearing water types systems on bacterial populations in the trout GIT lumen and mucosa. *Proteobacteria* and *Firmicutes* were the two most abundant phyla in both the rainbow trout GIT and water samples. Diet is typically observed to be an important variable shaping the microbiota of the GIT, but no significant variation in the GIT microbiota was observed in our study with respect to diet. Instead water system appeared a more important factor in shaping rainbow trout microbiota. Our results suggest that rearing water enriches distinct microbiota in the GIT of rainbow trout. We suggest a more detailed study looking at the effect of specific ingredients that could be utilized to influence the water microbial community, particularly as it relates to those bacteria associated with certain diseases in freshwater fishes.

## Supporting information

S1 Fig Bacterial composition that are significantly different at the genus level between luminal (L) and mucosal (M) GIT sections. (DOCX)

S2 Fig Bacterial composition that are significantly different at the genus level between mucosal (M) and water (W) samples. (DOCX)

S3 Fig Bacterial composition that are significantly different at the genus level between luminal (L) and water (W) samples. (DOCX)

S1 Table Major core bacteria taxa across the five taxonomic lineages in rainbow trout fed animal and plant protein diets. (DOCX)
