# Supplementary material for: Water system is a controlling variable modulating bacterial diversity of gastrointestinal tract and performance in rainbow trout
Source: PLoS One. 2018 Apr 17;13(4):e0195967. doi: 10.1371/journal.pone.0195967 (PMC5903623; doi:10.1371/journal.pone.0195967)
Supplement: S1 Fig — (DOCX) [file pone.0195967.s001.docx]

**Supplementary materials**
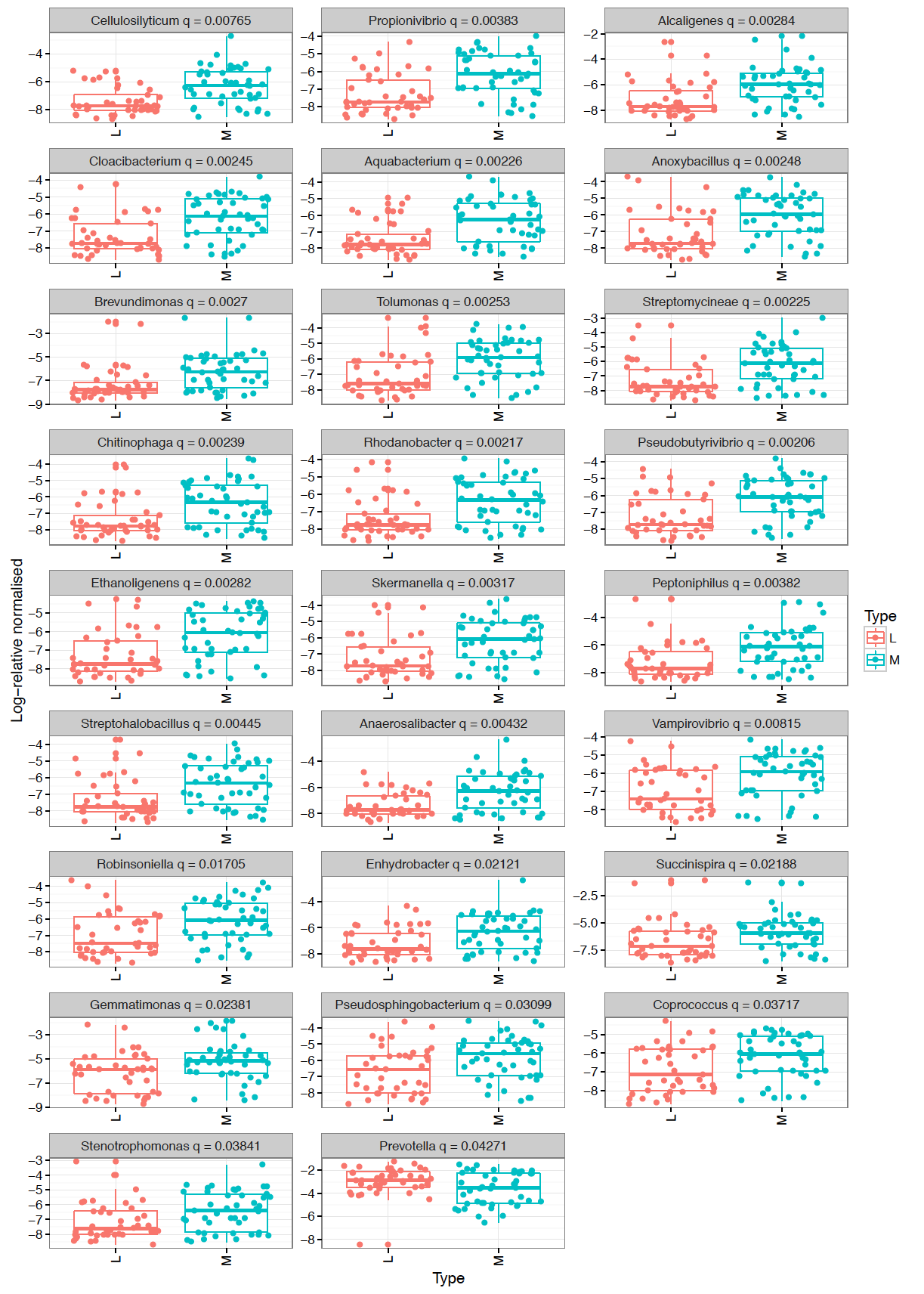

S1. Bacterial composition that are significantly different at the genus level between luminal (L) and mucosal (M) GIT sections.
