# Supplementary material for: Water system is a controlling variable modulating bacterial diversity of gastrointestinal tract and performance in rainbow trout
Source: PLoS One. 2018 Apr 17;13(4):e0195967. doi: 10.1371/journal.pone.0195967 (PMC5903623; doi:10.1371/journal.pone.0195967)
Supplement: S1 Table — (DOCX) [file pone.0195967.s004.docx]

Table S1. Major core bacteria taxa across the five taxonomic lineages in rainbow trout fed animal and plant protein diets.

| Phylum | Class | Order | Family | Genus |
| --- | --- | --- | --- | --- |
| *Firmicutes* | *Actinobacteria* | *Clostridiales* | *Porphyromonadaceae* | *Paludibacter* |
| *Proteobacteria* | *Clostridia bacteroisia* | *Bacteroidales* | *Lachnospiraceae* | *Flavobacterium* |
| *Bacteroidetes* | *Gammaproteobacteria* | *Flavobacteriales* | *Flavobacteriaceae* | *Prevotella* |
| *Actinobacteria* | *Alphaproteobacteria* | *Lactobacillales* | *Prevotellaceae* | *Aeromonas* |
| *Acidobacteria* | *Bacilli* | *Rhizobiales* | *Aeromonadeae* | *Parasporobacterium* |
| *Fusobacteria* | *Flavobacteria* | *Aeromonas* | *Streptococcaceae* | *Lactococcus* |
|  | *Negativicutes* | *Actinobacteridae* | *Acticetalesnomy* | *Clostridium XiVa* |
|  | *Episilonproteobacteria* | *Bacillales* | *Clostridiaceae* | *Sarcina* |
|  | *Fusobacteria* | *Selenomonadales* | *Rhizobiaceae* | *Arcobacter* |
|  | *Acidobacteria* | *Campylobacterales* | *Campylobacteraceae* | *Bacteroides* |
|  |  | *Pseudomonadales* | *Bacteroidaceae* | *Clostridium sensu* |
|  |  | *Xanthomonadales* | *Moraxellaceae* | *Streptococcus* |
|  |  | *Sphingomonadales* | *Sphingomonadaceae* | *Sphingomonas* |
|  |  | *Fusobacteriales* | *Pseudomonadaceae* | *Steroidobacter* |
|  |  | *Gp4* | *Acidaminococccaceae* |  |
|  |  |  | *Planococcaceae* |  |
|  |  |  | *Bacillaceae* |  |
|  |  |  | *Sinobacteraceae* |  |
